# Sepsis in the burn patient: a different problem than sepsis in the general population

**DOI:** 10.1186/s41038-017-0089-5

**Published:** 2017-08-08

**Authors:** David G. Greenhalgh

**Affiliations:** 1 Shriners Hospitals for Children Northern California, 2425 Stockton Blvd., Sacramento, CA 95817 USA; 2 Firefighters Regional Burn Center at University of California, Davis, USA; 3 Department of Surgery, University of California, Davis, USA

**Keywords:** Sepsis, Septic shock, Infection, Inflammation, Burns, Pediatric, Multiple organ dysfunction syndrome

## Abstract

Sepsis has recently been defined as “life-threatening organ dysfunction caused by a dysregulated host response to infection”. A great amount of effort has been made to develop early treatments for sepsis through the Surviving Sepsis Campaign. There are similar but slightly different recommendations for the treatment of sepsis in the pediatric population. These international efforts have led to earlier diagnosis and treatments for sepsis that have led to improvements in survival. Sepsis is also the leading cause of death in the burn patient but most clinical sepsis studies have excluded burns. The reason for the exclusion is that the sepsis found in burn patients is different than that of the general population. The early treatment strategies, such as those directed by the Surviving Sepsis Campaign, focus on patients presenting to hospitals with recent signs of infection. Burn patients lose their primary barrier to infection, the skin, and thus the risk of infection persists as long as that barrier is absent. Efforts have been made to define sepsis, septic shock and infection in the burn population but there is constant need for revisions. One focus of this review is to discuss the differences in burn sepsis versus sepsis of the general population. Children often have profound responses to sepsis but can also make remarkable recoveries. This review will also explore problems specific to pediatric burns. The treatment of burns requires a continuous vigilance to watch for the subtle early signs of sepsis and then expeditious initiation of aggressive therapy. Strategies covering optimal management of pediatric burn sepsis will also be summarized.

## Background

The primary cause of death in burn patients who survive initial burn shock resuscitation is from multiple organ dysfunction syndrome (MODS), which is a direct response to sepsis. The same is true for all patients admitted to an intensive care unit. Unfortunately, there has been only moderate improvement in survival in patients suffering from sepsis over the past several decades. Because of these dire statistics, there has been an effort to improve the speed of diagnosis and shorten the time for treatment of sepsis. All sepsis trials have excluded burn patients for several reasons. Burn patients have lost the primary barrier to infectious invasion, their skin. In addition, patients with extensive burns develop a profound hypermetabolic response that persists for months. They are at risk for sepsis and MODS at least as long as the wounds remain open. Despite these issues, there have not been formal efforts to improve the diagnosis and treatment of sepsis in burn patients. The diagnosis and treatment of sepsis in pediatric burn patients has received even less attention. The goal of this review is to describe basic concepts of sepsis, and describe the specifics of burn sepsis in both adults and children.

## Review

### Pathophysiology of sepsis

There is a great deal known about the pathophysiology of sepsis. Sepsis is an extreme response to inflammation. One of the best descriptions of inflammation is provided by Medzhitov who defined it as “an adaptive response for restoring homeostasis in response to some form of stress” [1]. Resident macrophages act as “sentinels” that detect and respond to any disturbance in the tissues. A very mild insult, such as an invasion of a few bacteria can be handled solely by these macrophages. With a minor insult such as for a small injury, the macrophages “call for help” by signaling for other leukocytes to help fight infection or for fibroblasts to isolate the infection and heal the wound. Communication for this “inflammatory pathway” can be divided into four categories: *“inducers, sensors, mediators and effectors”*. Inducers are signals that initiate the inflammatory response. Inducers include molecules released from bacteria and viruses (pathogen-associated molecular patterns—PAMPs) or from damaged cells (damage-associated molecular patterns—DAMPs). The classic PAMP is lipopolysaccharide (LPS) from gram-negative bacterial cell walls. Viral DNA or RNA, or urate crystals are also PAMPs. When a cell is damaged, it releases mitochondria and other molecules (such as high mobility box group 1—HMBG1) that will also initiate an inflammatory response. Sensors are the cell receptors (pathogen recognition receptors—PRRs) that recognize PAMPs or DAMPs. The classic sensor is “toll-like receptor-4 (TLR4)” which binds LPS to initiate the inflammatory response. When an inducers binds to a sensor intracellular signaling is initiated through multiple pathways leading to gene expression to produce mediators that are used for cell signaling. The classic mediators are “cytokines” such as tumor necrosis factor-α (TNF-α), interleukin-1 (IL-1) and interleukin-6 (IL-6). Finally, effectors are the cells, tissues and organs that respond to the release of effectors.

With a mild insult, low levels of cytokines are produced and there is just a local response. The localized inflammatory response may lead to an abscess or simply initiate the healing of the wound. Once the injury reaches a certain threshold size, such as after a 15% total body surface area (TBSA) burn, cytokines “spill” into the systemic circulation. Circulating cytokines are then detected in the brain, especially the hypothalamus, and the entire focus of the body’s organs is redirected to deal with the infection. The pituitary gland signals for the release of catecholamines and glucocorticoids from the adrenal gland leading to tachycardia, tachypnea and an increase in metabolic rate. The increase in metabolic rate creates a low grade febrile state that may persist as long as the signaling persists. To create fuel for fighting infection and healing wounds, there is preferential breakdown of muscle to convert amino acids to pyruvate. The liver redirects its energy to create acute phase proteins such as C-reactive protein [2]. The term for this total body change is systemic inflammatory response syndrome (SIRS). SIRS that is associated with an infection is called sepsis.

Sepsis has profound effects on all parts of the body. Just like for any injury, cytokines initiate capillary leakage that affects most of the capillary beds. There are intraluminal receptors on endothelial cells that turn on the Rho pathway to open the spaces between cells to create this leak [3]. The endothelial cells also upregulate the inflammatory response [4]. In addition, there is a shift to a pro-coagulation state and that, along with capillary damage, leads to platelet adhesion and consumption of coagulation factors. One of the earliest signs of sepsis is a drop in platelet count [5–7]. There are small areas of capillary bed thrombosis that leads to hypoperfusion. The consumption of clotting factors and platelets will ultimately result in disseminated intravascular coagulopathy (DIC). In response to hypoxia, cells produce nitric oxide (NO) that leads to the generalized decrease in systemic vascular resistance that is typically seen in sepsis. Therefore, there is a blend of both perfused and unperfused capillary beds. The unperfused regions build up lactic acid levels, another early sign of sepsis. The combination of an extensive capillary leak along with poor perfusion will ultimately lead to relative intravascular hypovolemia and hypotension—the manifestations of septic shock.

Poor perfusion ultimately takes its toll on organ function. Organ dysfunction is a key indicator of sepsis, and when multiple organs fail, the patient is said to have MODS [8, 9]. If the lungs are the target of injury, the leaking fluid interferes with the transfer of oxygen from the alveoli to the capillaries. The fluid shift can be rapid and profound leading to acute respiratory distress syndrome (ARDS). As fluid passes into the interstitial space, the intravascular deficiency is manifested by rising blood urea nitrogen (BUN). If there is low flow to the renal tubules in association with toxic molecules the patient will develop acute tubular necrosis (ATN). Similar damage to the liver will lead to an increase in serum liver enzymes and bilirubin. The patient often becomes confused and agitated. Despite a low systemic resistance and resulting high cardiac output, the heart eventually becomes less efficient. As more organs fail, the odds that the patient will fail to recover increase. As of yet, there are no methods of blocking the processes of MODS other than to reverse the inciting cause. Even after treating the inciting cause, the mortality from MODS remains very high.

Sepsis has profound effects on the immune system. As a simplification, the immune system is divided into two systems—the innate system (existing in all multicellular organisms) that consists of pathogen recognition receptors (as described above) and the adaptive system (existing in vertebrates except fish lacking mandibles) that involves lymphocyte activation [10–12]. In the past, sepsis was described as a two stage phenomenon, an initial pro-inflammatory response leading to SIRS that was followed by a “compensatory anti-inflammatory response syndrome” (CARS) that was characterized by an immunosuppressive state [13, 14]. During the CARS period the patient was predisposed to new infections and the development of multiple organ failure. If normal immune function did not return the patient usually expired. In simple terms, one could consider that the pro-inflammatory state was dominated by the innate immune system and immunosuppression was dominated by the adaptive system. In addition, a secondary insult, such as a subsequent infection, would precipitate a more profound SIRS or CARS response. This was described as a “second hit” phenomena [15, 16]. The sequential pro-inflammatory followed by anti-inflammatory process has been challenged by a recent multi-center study that evaluated gene expression in patients admitted with sepsis. This study demonstrated that there was simultaneous up-regulation of both pro and anti-inflammatory genes that they described as a “genomic storm”. They also did not find a “second hit” that led to patient decline, but instead, there was a lack of improvement in adverse gene responses that would predispose the patient to a poor outcome. They suggest that the ultimate cause of MODS was from a persistent hyper-inflammatory state [17].

Another group has agreed with the simultaneous pro- and anti-inflammatory hyperactivity during early sepsis but they noted that instead of a pro-inflammatory state, there are studies that demonstrate a persistent immunosuppressive state that is associated with MODS [18, 19]. The dominant immunosuppression that occurs in prolonged sepsis is common to many other critical illnesses, a diagnosis that has fit into the general category of “chronic critical illness” (CCI) [20, 21]. With a profound injury or infection, neutrophils are consumed at a rate that is above the usual steady state of myelopoiesis. Since there is a high demand for more neutrophils, macrophages and dendritic cells, the hematopoietic system has to change to a state of “emergency granulopoiesis” [22] to produce enough of these inflammatory cells. Since the bone marrow has a limited productive capacity, there is a “reciprocal” reduction in lymphopoiesis and hematopoiesis [23, 24]. The result is the lymphopenia and anemia that is common with chronic illness. The other consequence is that there is a reduction in the differentiation of immature myeloid cells into mature cells that can respond to innate immunity signals. Therefore, there is an upregulation in myeloid-derived suppressor cells (MSDCs) that contribute to a dominant immunosuppressive state [25]. The group at University of Florida has called the chronic illness that persists in prolonged sepsis as “persistent inflammation-immunosuppression and catabolism syndrome (PICS)” [26]. This term fits with the wasting and repeated sepsis that is associated with MODS. Whether the late effects of sepsis are one of pro-inflammation or immunosuppression is probably not of much importance. It is likely that both pathways contribute to the high mortality of sepsis.

### New definitions of sepsis

Over the past two and a half decades there has been a tremendous effort to develop standardized approaches to the diagnosis and treatment of sepsis. The diagnoses were based on signs of SIRS: temperature >38 °C or <36 °C, heart rate >90 beats per minute, respiratory rate >20 breaths per minute or maintenance of PaCO_2_ < 32 mmHg, or white blood count >12,000/mm^3^ or 4000/mm^3^ or left shift defined as >10% bands. Two or more of these signs were considered to diagnose SIRS [8]. When there is a culture positive infection, pathologic tissue source identified or a clinical response to antibiotics in addition to the above SIRS signs, the patient was considered to have sepsis. In the past, there was also a term called severe sepsis which was considered sepsis in combination with MODS. Septic shock was defined as sepsis associated with persistent hypotension (mean arterial pressure [MAP] <65 mmHg) despite adequate fluid resuscitation and/or lactate >4 mmol (36 mg/dl).

In an attempt to standardize definitions and even more importantly develop universal treatment guidelines, clinicians met to develop the Surviving Sepsis Campaign [27, 28]. This campaign was an effort to create strategies for the early diagnosis and set the standards for early treatment of sepsis. They developed “bundles” of treatment guidelines that needed to be followed within certain timelines in order to improve the outcomes of sepsis. Their efforts have demonstrated that the earlier the recognition and treatment of sepsis the better the outcome [29–31].

It is important to realize that every treatment guideline needs to be evaluated and updated on a regular basis. Within the last year, new sepsis definitions have been created. First, the “Third International Consensus Definitions for Sepsis and Septic Shock (Sepsis-3)” met to create new definitions [32–34]. This consensus group consisted of 19 members from the Society of Critical Care Medicine and the European Society of Intensive Care Medicine. They reviewed several databases (University of Pittsburgh Medical Center, Kaiser Permanente Northern California, Veterans Administration Ann Arbor Health System, Washington State Department of Health, King County Emergency Medical Services, University of Washington, and Jena University Hospital) “to evaluate the validity of clinical criteria to identify patients with suspected infection who are at risk for sepsis”. After reviewing nearly 150,000 patients, they created new definitions for sepsis and septic shock, and eliminated the term “severe sepsis”. They also created the clinical criteria required to identify those patients at risk for sepsis or septic shock.

#### Sepsis-3 definitions [32–34]

***Sepsis*** *– Sepsis is life-threatening organ dysfunction caused by a dysregulated host response to infection*

***Septic Shock*** *– Septic shock is a subset of sepsis in which underlying circulatory and cellular/metabolic abnormalities are profound enough to substantially increase mortality*

#### Sepsis-3 clinical criteria for identification [32–34]

***Sepsis*** *– Suspected or documented infection and an acute increase of* *>* *2 Sequential (Sepsis-related) Organ Failure Assessment (SOFA) points* (SOFA score [35] is a proxy for organ dysfunction)

***Septic Shock*** – *Sepsis* and *vasopressor therapy* needed to elevate *MAP* *>* *65* mmHg and *lactate > 2 mmol/L* (18 mg/dL) *despite adequate fluid resuscitation*

These new definitions and indicators have based on clinical data and simplify the diagnosis of sepsis.

In early 2017, the third and newest version of Surviving Sepsis Campaign (“Surviving Sepsis Campaign 2016”) was published [36]. This product is the result of another consensus committee of 55 experts representing 25 international organizations and it did include the concepts from the Sepsis-3 definitions. They divided experts into panels to review the literature of several different topics. They then assessed the quality of evidence based on the Grading of Recommendations Assessment, Development, and Evaluation (GRADE) system. The GRADE system is a method that assesses the quality of evidence from “high” to “very low”. In addition, they made recommendations that were considered “strong”, “weak”, or “best practice” [37–39]. They created a document that can be utilized as a standardized strategy for the early treatment for all aspects of sepsis and septic shock (Table 1). The entire recommendations cannot be covered in this text, but key points can be made. Initial resuscitation should begin with giving 30 ml/kg of intravenous crystalloid fluids within 3 h of diagnosis. After that, bolus, fluids should be based on reassessment of the fluid status. The target MAP should be >65 mmHg with the goal to lower lactate levels to normal levels. Cultures should be obtained prior to starting any antibiotics but empiric broad-spectrum antibiotics that cover likely pathogens should be started within one hour of diagnosis. Once pathogens are identified, antibiotic coverage should be narrowed to cover that organism. They also stated that 7–10 days is adequate for most patients. The goal for all patients is to de-escalate antibiotics as soon as possible. Source control, the draining of abscesses, and removal of infected tissue or devices should also be performed as early as possible. The first choice for vasopressors is norepinephrine but vasopressin and epinephrine are second choices. Dobutamine is the choice for improving cardiac output if the patient has adequate volume on board. There are also recommendations for ventilator support that are consistent with the “ARDSNet” studies [40]. The many other recommendations are summarized in the table. Despite these efforts, sepsis continues to fill intensive care units and be a major contributor to mortality. Adherence to these guidelines is an important step in improving the treatment of sepsis and septic shock.

**Table 1:** Surviving Sepsis Campaign 2016 Recommendations [36]

A. Initial resuscitation
1. Sepsis and septic shock are emergencies – treatment should start immediately
2. Hypoperfusion – give 30 ml/kg IV crystalloid within 3 h
3. After fluids, additional fluids depend on reassessment of hemodynamic status
4. Further hemodynamic assessment (cardiac function) if clinical exam not helpful
5. Prefer dynamic over static variables be used to assess hemodynamic status
6. Target MAP 65 mmHg when using pressors
7. Aim to lower lactate to normal levels
B. Screening for sepsis and performance improvement
1. Hospitals should have a performance improvement program for sepsis – including sepsis screening
C. Diagnosis
1. Appropriate cultures should be obtained before starting antimicrobial therapy
D. Antimicrobial therapy
1. Start IV antimicrobials within one hour of diagnosis of sepsis and septic shock
2. Start empiric broad-spectrum therapy to cover likely pathogens
3. Narrow coverage once pathogens are identified and sensitivities are established, or clinical improvement
4. Recommend against sustained antimicrobial prophylaxis in patients with severe inflammatory states (burns, pancreatitis)
5. Optimize dosing based on pharmacokinetic and pharmacodynamic principles
6. Start empiric combination therapy (at least two of different classes) aimed at likely organisms for septic shock
7. Do not use combination therapy for other serious infections (sepsis, bacteremia)
8. Do not use combination therapy for neutropenic sepsis
9. De-escalate combination therapy within first few days in response to improvement for septic shock
10. Treatment for 7–10 days is adequate for most infections causing sepsis/septic shock
11. Longer courses are appropriate in patients with slow response, undrainable foci of infection, bacteremia with *S. aureus*, some fungi or viruses, or immunologic deficiencies
12. Shorter courses are appropriate for patients with rapid resolution following source control
13. Daily assessment for de-escalation
14. Procalcitonin can be used to shorten therapy
15. Procalcitonin can be used to support discontinuation of antibiotics
E. Source control
1. Search for a diagnosis that can be treated with source control (i.e., abscess, infected wound)
2. Remove intravascular access devices that could be a cause of sepsis as soon as possible (change lines)
F. Vasoactive medications
1. Norepinephrine is the first choice for vasopressor
2. Add vasopressin (up to 0.03 units/min) or epinephrine to norepinephrine next
3. Use dopamine only in highly selected patients (low risk for tachyarrhythmias and bradycardia)
4. Do not use dopamine for renal protection
5. Use dobutamine in patients with persistent hypoperfusion despite adequate volume status and use of vasopressors
6. Arterial lines should be placed if on vasopressors
G. Fluid therapy
1. Continue fluid challenges as long as hemodynamic factors improve
2. Use crystalloids as fluid of choice for initial resuscitation and subsequent volume replacement
3. Use balanced crystalloids or saline for fluids
4. Add albumin to crystalloids when patients require large volumes
5. Do not use hydroxyethyl starches
6. Crystalloids are preferred over gelatins
H. Corticosteroids
1. Do not use steroids if fluids and vasopressors are effective. If not, IV hydrocortisone at 200 mg/day
I. Blood products
1. Transfuse blood only when hemoglobin <7.0 mg/dL (except in extenuating circumstances – myocardial ischemia, severe hypoxemia, acute hemorrhage)
2. Do not use erythropoietin for anemia
3. Do not use fresh frozen plasma to correct clotting abnormalities in the absence of bleeding or planned invasive procedure
4. Transfuse platelets when <10,000/mm^3^, and when <20,000 mm^3^ if at risk for bleeding, ≥50,000 mm^3^ for active bleeding, surgery or invasive procedures
J. Immunoglobulins
1. Do not use IV immunoglobulins for sepsis/septic shock
K. Blood purification
1. No recommendation about blood purification
L. Anticoagulants
1. Do not use antithrombin for sepsis/septic shock
M. Mechanical Ventilation (for sepsis-induced ARDS in adults)
1. Target tidal volume of 6 mL/kg predicted body weight (not 12 mL/kg)
2. Use upper limit goal for plateau pressures of 30 cm H_2_O
3. Use higher PEEP over lower PEEP
4. Use recruitment maneuvers
5. Use prone position over supine if P/F <150
6. Do not use high-frequency oscillatory ventilation
7. No recommendation about noninvasive ventilation
8. Use neuromuscular blocking agents for ≤48 h if P/F < 150
9. Use a conservative fluid strategy if no hypoperfusion
10. Do not use β-2agonists if no bronchospasm
11. Do not use a pulmonary artery catheter for sepsis-induced ARDS in adults
12. Use lower tidal volumes in sepsis-induced respiratory failure without ARDS
13. Elevate the head of bed to 30°–45° in ventilated patients
14. Use spontaneous breathing trials in ventilated patients
15. Use weaning protocols in patients who can tolerate weaning
N. Sedation and Analgesia
1. Minimize continuous or intermittent sedation in ventilated patients
O. Glucose control
1. Use a protocol for glucose control when two consecutive glucoses >180 mg/dL (not 110)
2. Monitor glucoses every 1–2 h until stable, then every 4 h if on insulin infusion
3. Interpret point-of-care glucoses with caution
4. Use arterial over capillary blood if arterial line present
P. Renal replacement therapy
1. Use either continuous or intermittent renal replacement therapy
2. Use continuous renal replacement therapy if hemodynamically unstable
3. Do not use renal replacement therapy just for increased creatinine or oliguria without other definitive indications for dialysis
Q. Bicarbonate therapy
1. Do not use sodium bicarbonate with lactic acidemia with pH ≥ 7.15
R. Venous thromboembolism prophylaxis
1. Use pharmacologic prophylaxis (UFH or LMWH) in the absence of contraindications
2. Use LMWH rather than UFH
3. Combine pharmacologic prophylaxis and mechanical prophylaxis whenever possible
4. Use mechanical prophylaxis when pharmacologic prophylaxis is contraindicated
S. Stress ulcer prophylaxis
1. Give stress ulcer prophylaxis to patients at risk for GI bleeding
2. Use either proton pump inhibitors or histamine-2 receptor antagonists
3. Do not use stress ulcer prophylaxis in patients without risk factors for GI bleeding
T. Nutrition
1. Do not use parenteral feedings if enteral feedings possible
2. Do not provide parenteral nutrition for the first 7 days if enteral feedings not possible (advance enteral feedings as tolerated)
3. Start early enteral feedings if possible
4. Start early trophic/hypocaloric or early full feedings (advance as tolerated)
5. Do not use omega-3 fatty acids
6. Do not check routine gastric residual volumes (but check if feeding intolerance or high risk for aspiration – applies to nonsurgical patients)
U. Setting goals of care
1. Goals of care and prognosis should be discussed with the patient and families
2. Goals of care should be incorporated into treatment and end-of-life planning, using palliative care principles when appropriate
3. Address goals of care as early as feasible, but no later than 72 h after ICU admission

*IV* Intravenous, *MAP* Mean arterial pressure, *S. aureus* Staphylococcus aureus, *dl* deciliter, *ARDS* acute respiratory distress syndrome, *PEEP* positive end expirato ry pressure, *P/F* PaO2/FIO, *UFH* unfractionated heparin, *LMWH* Low molecular weight heparin, *GI* gastrointestin al, *ICU* intensive care unit

### Sepsis in the pediatric patient

Sepsis in the pediatric population should not be considered equal with sepsis observed in adults. There are many differences in treating an infant than an adult and especially a geriatric patient. While this review will not focus on the many differences for routine pediatric care and that for adults, there have been similar efforts to improve the optimal care of pediatric and neonatal sepsis. The latest “clinical practice parameters to support pediatric and neonatal septic shock” was published in 2017 [41]. The differences between adults and pediatrics will be summarized here. This review will not, however, cover neonatal septic shock. As for adults, strategies that provide both rapid diagnosis and early treatment protocols improve survival in pediatric and neonatal sepsis [42, 43]. In addition, the pediatric guidelines provide excellent principles, or as they call them, “home-grown bundles”, that apply for all age groups. All facilities should develop sepsis bundles include the following key components:

1. A recognition bundle containing a trigger tool for rapid identification of patients with septic shock
2. A resuscitation and stabilization bundle for early treatment
3. A performance bundle to monitor, improve, and sustain adherence

Utilizing these principles has led to improved survival for patients with sepsis of all ages.

For adults, the predominant cause of mortality is “vasomotor paralysis” [44] that is dominated by myocardial dysfunction with decreased ejection fraction. The patient compensates by increasing heart rate and ventricular dilation. If they do not adapt by increasing heart rate or ventricular dilation they have a high mortality. In addition, adults have a very low systemic vascular resistance (SVR) during sepsis. Pediatric septic shock is usually associated with profound hypovolemia but the response to fluid is often different than that of adults. Mortality for children is more often associated with low cardiac output than low SVR. The goal in the pediatric population is to obtain a cardiac index of 3.3–6.0 L/min/m^2^. In adults, there is a defect in oxygen extraction in the tissues, but for pediatrics, there is a defect in oxygen delivery.

There are clinical signs that are more important for the diagnosis of sepsis in pediatrics. The key findings are hypothermia or hyperthermia, altered mental status, peripheral vasodilation for “warm shock”, capillary refill <2 s (vasoconstriction) for “cold shock”. The threshold heart rates for concern are outside the following ranges: 110–160 for an infant, 90–160 for an infant (<2 years) and 70–150 for a child (7 years of age). The blood pressure measurement that triggers a reaction is based on perfusion pressure, which equals MAP minus central venous pressure (CVP). The trigger for action based on perfusion pressure is when the value lower than the following formula, perfusion pressure = MAP-CVP = (55 + [age × 1.5]). Values below 55 for the neonate, 58 for the infant (2 years), and 62 for the child (7 years) should prompt rapid attempts to improve perfusion pressures by providing fluids, and if unresponsive, vasopressors.

The pediatric guidelines [41] are provided here but in principle they apply to patients of all ages who present with shock. The diagnosis should be made within 5 min and the initial treatment bundle should be initiated within 15 min. A bolus of 20 ml/kg of crystalloid or 5% albumin should be initiated within 15 min and a vasopressor started within 60 min if there is no response to the fluid challenge of a total of 60 ml/kg. The preferred vasopressor for pediatric septic shock is epinephrine but dopamine or norepinephrine may also be used. The vasopressor choice is different than that suggested for adults. Norepinephrine is the drug of choice for adults and dopamine has fallen out of favor. For all ages, broad spectrum antibiotics should be initiated within 60 min after obtaining blood cultures. Dobutamine is also acceptable for all ages when pure inotropic support is needed. Another difference for children is that there is more support for using vasodilators, such as nitroprusside or nitroglycerine when low cardiac output is associated with high SVR. The other option for pediatric septic shock is to use type III phosphodiesterase inhibitors such as milrinone or inamrinone since they increase cardiac output and lower SVR. Finally, extracorporeal support (extracorporeal membrane oxygenation) is more commonly used and is more successful in the pediatric population than for adults.

### Sepsis in the burn patient

While there have been tremendous efforts to improve the early diagnosis and treatment of sepsis in the general population, there has been very little progress in managing sepsis in burn patients. It is important to remember that there are several differences between sepsis in the general population and sepsis found after a burn injury. Burn patients lose the first barrier to infection—their skin. The burn patient is continuously exposed to inflammatory mediators as long as the wound remains open. When there are extensive burns the exposure to pathogens will persist for months. Therefore, all burns >15–20% TBSA will have a persistent “SIRS” that persists for months after the wound is closed. Because of this hypermetabolic response, these patients have persistent tachycardia, tachypnea, leukocytosis, and reset their normal temperature to around 38 °C. In other words, at baseline burn patients always have the signs used to diagnose sepsis in the general population.

Because burn patients have persistent SIRS they are always excluded from any sepsis trial, including Sepsis-3 [32–34] and Surviving Sepsis Campaign 2016 [36]. In an effort to create definitions that apply to burn patients, members of the American Burn Association held a Consensus Conference in 2007. Experts in burns and sepsis reviewed the literature and presented definitions for several topics related to sepsis and infection in burns. A consensus was then obtained from the group and the results were published in 2007 [45]. First of all, everyone agreed that all patients with burns >20% TBSA have SIRS. The definition for sepsis in burns was defined as the following:

Sepsis: the presence of three or more of the following criteria:

- Temperature >39 °C or <36.5 °C
- Progressive tachycardia >110 beats per minute
- Progressive tachypnea >25 breaths per minute or minute ventilation >12 L/min
- Thrombocytopenia <100,000/mcl (does not apply until 3 days after burn)
- Hyperglycemia in the absence of pre-existing diabetes mellitus
  - (Untreated plasma glucose >200 mg/dl or intravenous insulin >7 units/hr IV, significant resistance to insulin [>25% increase in insulin requirements over 24 h])
- Inability to continue enteral feedings >24 h
  - (Abdominal distension, enteral feeding intolerance [two times feeding rate], uncontrollable diarrhea [>2500 ml/day])

In addition, it is required that a documented infection is identified defined as:

- Culture positive infection or
- Pathologic tissue source identified or
- Clinical response to antimicrobials

The committee agreed to drop the term “*severe sepsis*”.

Septic shock: sepsis (as described above) plus shock-like hemodynamic parameters defined in the 2004 Surviving Sepsis Campaign.

The timing for the onset and thus the treatment is also different between burn patients and the sepsis that is typically seen in other populations. The patient targeted for the Surviving Sepsis Campaign is admitted from the community or the medical/surgical ward with new onset sepsis. These patients need a rapid diagnosis along with prompt initiation of treatment. Burn patients are admitted directly to the burn intensive care unit with hypovolemic shock from the initial injury. Sepsis rarely occurs within the first week but instead, occurs weeks to even months after the injury. As long as the wound remains open, the burn patient is at risk for developing sepsis. In addition, burn patients usually require long-term invasive central lines, urinary catheters and tracheal intubation. As long as these invasive devices are present, the risks for ventilator-associated pneumonia, urinary tract infections and central line associated bloodstream infections are substantially increased. In addition, the burn patient is profoundly immunosuppressed and is frequently colonized or infected with multiply resistant organisms. They are also prone to unusual infections such as viral or fungal infections. These patients require constant monitoring for subtle changes such as dropping platelet counts, increased fluid requirements, increased respiratory support, confusion, changes in the wound, and high fevers. In the patient with a massive burn, sepsis may occur multiple times, and the patient is never free from risk until the burn is discharged. Unfortunately, many of these massively burned patients have died with healed wounds. Therefore, the “bundles” used for the early treatment of the unburned population do not apply to the burn patient.

Because of these unique problems, there is a great need to develop early signs and symptoms of sepsis and septic shock in the burn population. There is no unanimity as to which signs or symptoms are of utility for the early diagnosis of burn sepsis. What should be the “trigger” to initiate care? Of even greater importance is the lack of early treatment “bundles” designed specifically for the early treatment of sepsis in the burn patient. Clearly, any delay in treatment will increase mortality. The clinical signs of sepsis in the burn patient are very subtle and are easily missed until profound septic shock is present. There are no guidelines as to the duration of antibiotic treatment and how to narrow antimicrobial coverage. The optimal hemodynamic support mechanisms are also not known. Questions, such as, should steroids be used, have no clear answers. What are the best methods for treating inhalation injury or acute respiratory distress syndrome? What is the best method for dealing with nutrition, glucose control or the hypermetabolic response? While there may be some overlap between the Surviving Sepsis 2016 suggestions and burn sepsis treatment, it is clear that there is a need for burn-specific guidelines. Hopefully, the burn community will develop guidelines specific to the burn patient.

### Sepsis in the pediatric burn patient

Very little has been published that specifically addresses sepsis in the pediatric burn patient. Pediatric burns were addressed in the American Burn Association Consensus Definitions [45] but the definitions essentially relied on an international pediatric consensus conference that defined sepsis and organ dysfunction in children [46]. In essence, the signs and symptoms of burn sepsis are similar to adults but one must remember that vital signs are age-dependent in the pediatric population. Clearly, younger children have higher heart and respiratory rates than adults. To adjust for normal variations the American Burn Association Consensus used diagnostic values suggested by the pediatric sepsis group – heart rate and respiratory rate two standard deviations above age-specific norms (or 85% age-adjusted maximal heart and respiratory rates). Thrombocytopenia was also adjusted for children to be less than two standard deviations below age-specific norms. For feeding intolerance, the value was set at >150 ml/h and for uncontrollable diarrhea the value was >400 ml/day. The values for septic shock were also defined as greater than two standard deviations below normal for age. These values must consider lower normal blood pressures along with higher heart and respiratory rates. In addition, the following signs were suggested: tachycardia with signs of decreased perfusion (this sign may be absent with hypothermia), decreased peripheral pulses compared with central pulses, altered alertness, flash capillary refill >2 s, mottled or cool extremities, and urine output <1 ml/kg.

The Galveston group published a review of over 800 pediatric burn patients who developed multiple organ failure using the DENVER2 definitions [47]. They found that respiratory failure tended to occur in the early phase of healing—starting at 5 days post-injury. Heart failure had the highest incidence throughout the entire hospital stay and hepatic failure increased throughout the stay. Hepatic failure was associated with a high mortality rate. They reported a low incidence of renal failure, but if it occurred, there was a high early mortality rate. As expected, failure of more than three organ systems was associated with a very high rate of death. There was a recent publication that described the etiologies associated with MODS in children [48]. They described factors associated with pediatric burns but they relied mostly on the Galveston study for their data.

Despite the lack of publications related to pediatric burn sepsis clinical experience can provide some important points. The subtle signs of sepsis such as high fever, dropping platelet count, decreased urine output, and hemodynamic changes are similar for adults and children. In most instances, sepsis tends to have an insidious onset but, on occasion, sepsis can have a very rapid course in children. In my experience, *Klebsiell*a may lead to profound septic shock within a few hours. Overall, children tend to have more profound responses to sepsis but despite being critically ill, they often bounce back and once healed, do very well. Once sepsis is expected, empiric broad-spectrum antibiotics should be started as soon as sepsis is suspected. Antibiotics should cover *Staphylococcus aureus* (*S. aureus*) (including methicillin-resistant *S. aureus*) and Gram-negative organisms. Routine blood, urine, and respiratory cultures should be obtained prior to starting antibiotics. Since central line infections are a relatively common cause of sepsis, all lines should be changed. The patient should have his or her wounds checked for any signs of infection. The diagnosis of wound infection is not made by culture but instead by finding changes in the appearance of the wound. The most profound wound infection is caused by *Pseudomonas aeruginosa* (*P. aeruginosa*) which, when invading the wound, creates purple to gray punched out lesions. The patient also develops rapid and profound shock. These wounds must be excised and the treatment with antibiotics specific for *P. aeruginosa* is required. Even if the wounds do not appear to be grossly infected, excision of the exposed areas with coverage with allograft seems to be helpful. Despite weeks of profound illness, persistence pays off since it is amazing how children can recover and lead normal lives.

## Conclusions

Sepsis in burn patients has many differences than that found in the unburned population. All burn patients require close monitoring for as long as the wound remains open. Pediatric burn patients may show more profound effects but aggressive therapy is worthwhile. The only burn-related definitions of sepsis, the American Burn Association Consensus Definitions [45], have recently been challenged as being less accurate than other diagnostic modalities [49, 50]. The challenges are welcome since the consensus definitions were never meant to be static. There is a great need for new efforts to develop accurate diagnoses that trigger rapid treatment with “burn sepsis bundles”. All new diagnostic criteria and treatments will need to be tested for their effectiveness. Hopefully, the burn community will develop new guidelines and sepsis bundles that lead to more improvements in the survival of burn patients.

## Abbreviations

ABA: American Burn Association
ARDS: Acute respiratory distress syndrome
ATN: Acute tubular necrosis
BUN: Blood urea nitrogen
CARS: Compensatory anti-inflammatory response syndrome
CCI: Chronic critical illness
CVP: Central venous pressure
DAMP: Damage-associated molecular pattern
DIC: Disseminated intravascular coagulopathy
dl: Deciliter
DNA: Deoxyribonucleic acid
GI: Gastrointestinal
GRADE: Grading of recommendation assessment development and evaluations
HMBG1: High mobility box group 1
ICU: Intensive care unit
IL-1: Interleukin-1
IL-6: Interleukin-6
IV: Intravenous
kg: Kilogram
LMWH: Low molecular weight heparin
LPS: Lipopolysaccharide
m: Meter
MAP: Mean arterial pressure
mcl: Microliter
mg: Milligram
min: Minute
ml: Milliliter
mm: Millimeter
mmHg: Millimeters mercury
mmol: Millimole
MODS: Multiple organ dysfunction syndrome
MSDC: Myeloid-derived suppressor cell
NO: Nitric oxide
*P. aeruginos*: *Pseudomonas aeruginosa*
P/F: PaO_2_/FIO_2_
PAMP: Pathogen-associated molecular pattern
PEEP: Positive end expiratory pressure
PICS: Persistent inflammation-immunosuppression and catabolism syndrome
PRR: Pathogen recognition receptor
RNA: Ribonucleic acid
*S. aureus*: *Staphylococcus aureus*
SIRS: Systemic inflammatory response syndrome
SOFA: Sequential (sepsis-related) organ failure assessment
SSC: Surviving sepsis campaign
SVR: Systemic vascular resistance
TBSA: Total body surface area
TLR: Toll-like receptor
TNF-α: Tumor necrosis factor - α
UFH: Unfractionated heparin
